# Novel hepatitis E like virus found in Swedish moose

**DOI:** 10.1099/vir.0.059238-0

**Published:** 2014-03

**Authors:** Jay Lin, Heléne Norder, Henrik Uhlhorn, Sándor Belák, Frederik Widén

**Affiliations:** 1 Department of Virology, Immunobiology and Parasitology (VIP), National Veterinary Institute (SVA), Uppsala, Sweden; 2 Department of Infectious Diseases/Section of Clinical Virology, Institute of Biomedicine, University of Gothenburg, Gothenburg, Sweden; 3 Department of Pathology and Wildlife Diseases, National Veterinary Institute (SVA), Uppsala, Sweden

## Abstract

A novel virus was detected in a sample collected from a Swedish moose (*Alces alces*). The virus was suggested as a member of the *Hepeviridae* family, although it was found to be highly divergent from the known four genotypes (gt1–4) of hepatitis E virus (HEV). Moose are regularly hunted for consumption in the whole of Scandinavia. Thus, the finding of this virus may be important from several aspects: (a) as a new diverged HEV in a new animal species, and (b) potential unexplored HEV transmission pathways for human infections. Considering these aspects, we have started the molecular characterization of this virus. A 5.1 kb amplicon was sequenced, and corresponded to the partial ORF1, followed by complete ORF2, ORF3 and poly(A) sequence. In comparison with existing HEVs, the moose HEV genome showed a general nucleotide sequence similarity of 37–63 % and an extensively divergent putative ORF3 sequence. The junction region between the ORFs was also highly divergent; however, two putative secondary stem–loop structures were retained when compared to gt1–4, but with altered structural appearance. In the phylogenetic analysis, the moose HEV deviated and formed its own branch between the gt1–4 and other divergent animal HEVs. The characterization of this highly divergent genome provides important information regarding the diversity of HEV infecting various mammalian species. However, further studies are needed to investigate its prevalence in the moose populations and possibly in other host species, including the risk for human infection.

## Introduction

Hepatitis E virus (HEV) belongs to the *Hepeviridae* family and is a small non-enveloped 7.2 kb capped single stranded, positive sense RNA virus. The genome consists of three open reading frames 1–3 (ORF1–3) and terminates with a poly(A)-tail (Tam *et al.*, 1991). The non-structural proteins involved in the viral replication are encoded by ORF1 in the two-thirds of the genome at the 5′ end (Koonin *et al.*, 1992). ORF1 is followed by a short section called the junction region (JR), which is predicted to fold into two RNA stem–loop structures critical for RNA replication (Cao *et al.*, 2010; Graff *et al.*, 2005; Huang *et al.*, 2007). The subsequent part of the genome is transcribed into a capped bi-cistronic subgenomic RNA (SgRNA) overlapping ORF2 and ORF3, with its 5′ terminal end positioned within the JR and terminating with poly(A) (Graff *et al.*, 2006). ORF2 encodes the viral capsid protein with HEV RNA binding, assembly, virion packaging and host cell attachment properties (Mori & Matsuura, 2011). ORF3 encodes a small accessory protein with an unclear function, but with the ability to interact with ORF2 and cell related proteins (Korkaya *et al.*, 2001; Zafrullah *et al.*, 1997). It is estimated that two billion of the world’s population is at risk of being infected by HEV (Aggarwal & Jameel, 2008) and four genotypes 1–4 (gt1–4) infect humans. Gt1 and gt2 are restricted to humans, and have a mortality rate of 0.5 %, or up to 28 % in pregnant women; the reason for this is still unknown (Aggarwal, 2011; Navaneethan *et al.*, 2008). Gt3 and gt4 have a wider host range with several animal species as hosts (Meng, 2011). HEV is transmitted mainly through the faecal–oral route and is an important causative agent of acute hepatitis in many developing countries, where it may cause large outbreaks (Okamoto, 2007), but is also present in developed countries with sporadic infections acquired locally within the country. These infections are caused by gt3 or gt4, often with unclear transmission routes (Purdy & Khudyakov, 2011). It seems that most HEV infections are asymptomatic and symptomatic cases alone only represent small part of the total (Kamar *et al.*, 2012). This is probably reflected by the high seroprevalence against hepatitis E in the general population. In Sweden, the seroprevalence is estimated to be 9 % (Olsen *et al.*, 2006), and between 5–30 % in other countries (Christensen *et al.*, 2008; Dalton *et al.*, 2007; Mansuy *et al.*, 2009). However, there may be more clinical cases of hepatitis E than notified, since many nonA–nonD hepatitis cases are not examined due to unawareness of endemic hepatitis E. A growing number of animal HEV variants have been identified in a wide range of non-porcine animals and some of them have highly divergent genomes compared to gt1–4. Pigs appear to be the main HEV reservoir of gt3–4, with high HEV prevalence in many countries. In Sweden it is estimated that 30 % of the 2–4 months old pigs are gt3 HEV infected (Widén *et al.*, 2011). Evidence for zoonotic transmission through ingestion of infected meat has been identified. Gt3–4 isolates with highly similar sequence identity were detected in patients and infected liver or meat from, for example, swine, wild boars and deer (Colson *et al.*, 2010; Li *et al.*, 2005; Meng, 2011; Takahashi *et al.*, 2004; Tei *et al.*, 2003).

Considering the limited understanding of the infection biology and host range of HEV, our aim was to investigate the existence of non-porcine HEV reservoirs for human infections and to better understand the relatedness of different HEV strains including the risk for humans that they may pose. The HEV prevalence in Swedish deer is unknown, but deer in other parts of Europe and Japan has been found to be HEV positive and been linked to human HEV infections (Pavio *et al.*, 2010; Takahashi *et al.*, 2004; Tei *et al.*, 2003; Tomiyama *et al.*, 2009). Here, we report for the first time the detection and genomic characterization of a new HEV in moose. The largest member of the deer family Cervidae, the moose (*Alces alces*), is regularly hunted and its meat consumed in Scandinavia. The knowledge that moose carry HEV opens additional unexplored transmission pathways of this virus to human populations. Based on the present investigation we propose that this newly detected moose virus is classified as a new member in the *Hepeviridae* family. Its infection biology should be studied in many aspects to clarify its potential as a zoonotic pathogen and shed more light on the increasingly complex *Hepeviridae* family.

## Results

### Detection and amplification of a HEV sequence from a moose sample

The knowledge that HEV can infect deer raised the possibility that moose might be infected with this virus. Indeed, when moose liver and kidney samples were screened by real-time PCR, one sample out of six was found to be HEV positive. This liver sample exhibited cycle threshold (C_t_) values of 33.4 and 34.6 with the Jothikumar *et al.* (2006) and Gyarmati *et al.* (2007) assays. This HEV positive liver sample was collected from a diseased three-year-old pregnant female moose. The carcass was severely decomposed and therefore unsuitable for histopathological examination. However, the following could be detected: cachexia (emaciation), anaplasmosis, acute heart muscle degeneration and haemorrhage, cysticercosis and ear mites.

A 383 nt sequence covering part of the RNA dependent RNA polymerase was obtained using primer pair 1 (Table 1). This region is frequently used for genotyping (Zhai *et al.*, 2006). According to nt blast, the closest match was gt3 with 88 % identity and covering only 41 % of the amplicon. To obtain more genome sequence, a series of PCRs were performed with three primer pairs 2–4 (Table 1). Primer pair 2 resulted in a 2.16 kb amplicon and primer pair 3 gave a 1.3 kb amplicon partially overlapped by the former amplicon. Primer pair 4 resulted in multiple products, and among these, only a 2.5 kb amplicon matched a HEV like sequence. By sequencing the amplicons, a 5.1 kb sequence was obtained corresponding to positions 2176–7227. All nt positions given for the sequences obtained in this study were referred to the Swedish swine gt3 HEV isolate SWX07-E1; accession number EU360977 (Xia *et al.*, 2008).

**Table 1.** Primers used for moose HEV genome amplification and sequencing

Primer	Primer ID		Nucleotide sequence	Product size (kb)	Position*(nt)	Reference
Primer sets						
1	ESP	(Forward)	CATGGTAAAGTGGGTCAGGGTAT	0.383	4248–4630	Zhai *et al.* (2006)
	EAP	(Reverse)	AGGGTGCCGGGCTCGCCGGA			
2	ESP	(Forward)	CATGGTAAAGTGGGTCAGGGTAT	2.16	4248–6410	Zhai *et al.* (2006)
	HE041R	(Reverse)	GCCAATGGCGAGCCGACAGTGAA			Mizuo *et al.* (2002)
3	HEV5979F	(Forward)	CGAGGAGGAGGCTACGTCTGGTCTGGTA	1.3	5979–7258	This study
	GenereRacer 3′Nest	(Reverse)	CGCTACGTAACGGCATGACAGTG			RACE kit (Invitrogen)
4	HEV108F	(Forward)	GCCTTGGCGAATGCTGTGGT	2.5–4.4	108/2176–4585	This study
	HEV4585R	(Reverse)	GGACTCCTTCGGAGCCTGCAGCGTCCAA			
Sequencing primers						
	M13F	(Forward)	CAGGAAACAGCTATGAC			TOPO XL kit (Invitrogen)
	HEV2491F	(Forward)	GGCTTGTCAATGCTGCAAACGCAGG		2467–2491	This study
	HEV2683F	(Forward)	GGCTCCGGTTGGCCTATATCGAGGC		2659–2683	This study
	HEV3499F	(Forward)	CCGTTCATGAGGCCCAGGGCGC		3478–3499	This study
	HEV4147F	(Forward)	CCGTCTTGGCCCTTATCCAGC		4127–4147	This study
	HEVF5	(Forward)	CTTTGGAAYACTGTTTGGAATATGG		4650–4674	Xia *et al.* (2008)
	HEV4835F	(Forward)	GCYTGTAYGCMGGCGWTGTC		4816–4835	This study
	HEV5162F	(Forward)	GAGGGAATAACATTCAGGATGCGC		5139–5162	This study
	HEV5597F	(Forward)	CGCCGACAGTACAATCTGTCAAC		5575–5597	This study
	HEV5967F	(Forward)	GGYTGGCGCTCYGTYGAGAC		5947–5967	This study
	HEV6345F	(Forward)	TGGCGGGCTCCCTACTGAGCTTGTGTCA		6318–6345	This study
	HEV7013F	(Forward)	CCAGTTCCTGCTGACGTGCTTGAGGC		6985–7013	This study
	HEV2491R	(Reverse)	CCTGCGTTTGCAGCATTGACAAGCC		2467–2491	This study
	HEVR3	(Reverse)	CGATATGCCGCCTCTAGCCTCTTGG		2671–2695	Xia *et al.* (2008)
	HEV3499R	(Reverse)	CCGTTCATGAGGCCCAGGGCGC		3478–3499	This study
	HEV3977R	(Reverse)	GACACTCTTACGATGGGCCGGTGCGG		3952–3977	This study
	HEV4147R	(Reverse)	CCGTCTTGGCCCTTATCCAGC		4127–4147	This study
	HEV4586R	(Reverse)	GGACTCCTTCGGAGCCTGCAGCGTCCAA		4559–4586	This study
	HEV5304R	(Reverse)	CCAACCACCACCTCCGCCGCCGCCCG		5279–5304	This study
	HEV5597R	(Reverse)	GTTGACAGATTGTACTGTCGGCG		5575–5597	This study
	HEV6105R	(Reverse)	GCGAAACTCCACCTGTAGGGC		6085–6105	This study
	HEV6841R	(Reverse)	CGGTCGTCGTGCCAGCCTGCCAATAG		6816–6841	This study
	M13R	(Reverse)	CAGGAAACAGCTATGAC			TOPO XL kit (Invitrogen)

* Positions based on the Swedish reference gt3, SWX07-E1 (EU360977.1) genome (Xia *et al.*, 2008).

### Moose HEV sequence properties

The moose HEV sequence contained three putative ORFs, which is characteristic for HEV. The 5′-region of the amplified sequence was located at the putative proline hinge region in the hypervariable region of ORF1 and extended through the partially overlapping ORF2–3 region to the poly(A) at the 3′ terminal end. The sequence was highly divergent from other described HEV variants and had the highest similarity to gt1–4 and unclassified wild boars (gt1–4-Uwb) group (Table 2).

**Table 2.** Comparative genome analysis of moose HEV and other HEV strains

	5.1 kb moose HEV sequence	Partial ORF1	ORF2	ORF3
	Genome position: 2176–7227*†‡	2176–5144*‡	5185–7152*‡	5168–5515*‡
	Actual size: 5057 nt	2966 nt	1968 nt	348 nt
	Sequence identity (%)	Sequence identity (%)	Sequence identity (%)	Sequence identity (%)
HEV variant (number of compared strains*)	Nt	Nt / aa	Nt / aa	Nt / aa
Genotype 1 (3)	61.9–62.9	62.8–63.4 / 66.6–67.1	65.7–66.1 / 72.4–73.0	53.3–53.9 / 30.3
Genotype 2 (1)	61.5	62.4 / 66.6	64.8 / 70.9	51.7 / 26.1
Genotype 3 (5)	62.9–63.1	61.8–62.8 / 65.5–66.5	66.3–67.6 / 72.9–74.0	52.8–55.0 / 29.4–31.1
Genotype 4 (3)	61.7–62.3	61.6–61.9 / 65.0–66.1	65.2–66.0 / 72.9–73.2	54.7–55.6 / 31.9–34.5
Rabbit (1)	60.9	59.8 / 63.5	65.7 / 72.0	54.7 / 34.5
Unclassified wild boars (Uwb) (2)	61.7–62.3	61.4–61.6 / 64.6–65.7	65.8–66.6 / 72.0–73.6	54.2–55.8 / 29.4–35.3
Rat (1)	52.1	52.9 / 53.4	55.7 / 56.0	32.8 / 17.0
Ferret (1)	52.6	54.0 / 54.5	55.3 / 54.9	35.8 / 16.8
Bat (1)	48.5	50.4 / 47.0	46.5 / 45.4	19.7 / 7.0
Avian (1)	46.1	50.2 / 45.4	44.7 / 41.6	23.3 / 11.0
Trout (1)	36.6	39.2 / 29.1	27.0 / 15.5	22.2 / 8.4

* Strain information is summarized in Table S1.

† Poly(A) sequence excluded.

‡ Positions of the Swedish gt3, SWX07-E1 (EU360977.1) genome.

### Characterization of the partial ORF1 sequence

The nt sequence 2176–5144 corresponding to the partial ORF1 sequence was analysed by blast and matched gt1 strains, but when translated to 987 aa, the blast search matched gt3 and gt4 strains. The moose HEV ORF1 stop codon (TAA) was shared with animal HEVs (rat, ferret, bat and trout) except for avian forms, which use TGA similar to the gt1–4-Uwb group.

### 

#### Characterization of the proline hinge region.

When the recovered moose HEV sequence was translated into aa, the first 79 N-terminal residues (position 717–795) corresponded to the proline hinge region. Although high sequence diversity was observed between the moose HEV and SWX07-E1, the number of prolines was similar, 19 compared to 21 (Fig. S1, available in the online Supplementary Material) indicating the functional importance of conserved numbers of prolines. This region has an unknown function, but may play a role in host adaptation (Purdy *et al.*, 2012).

#### Characterization of the X domain.

**Fig. 1.**
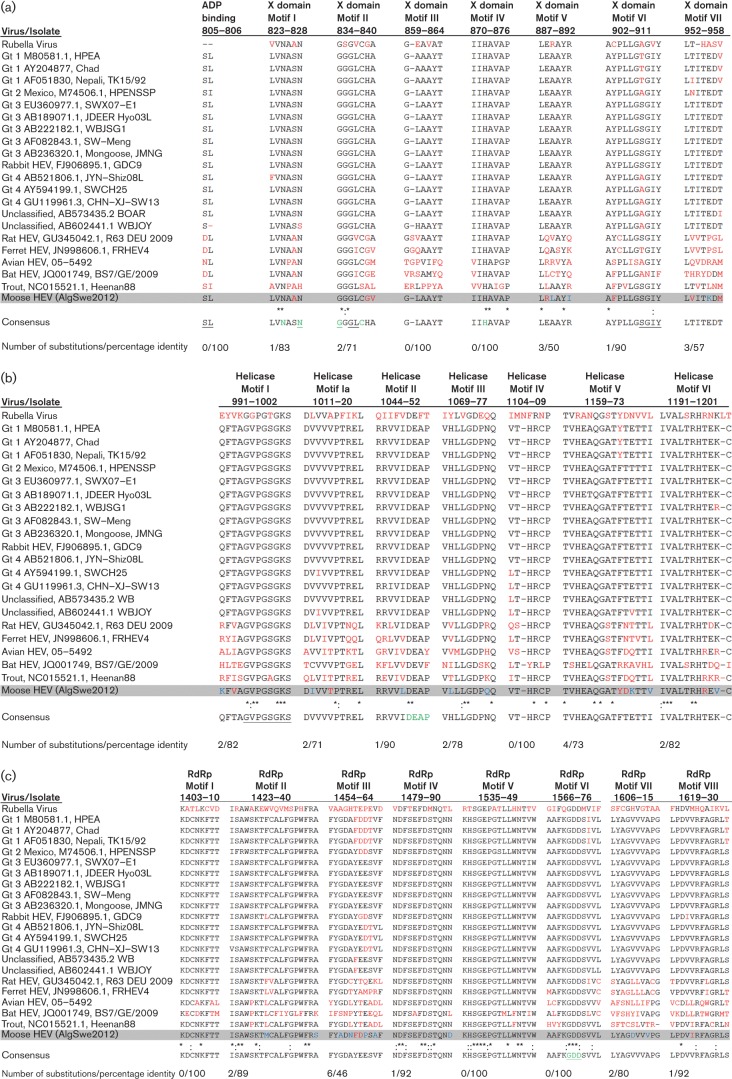
Comparison of aligned motifs within derived domains (Koonin *et al.*, 1992) from different HEV isolates and rubella virus. The moose HEV is highlighted in grey. Red letters represent non-consensus residues, asterisks mark identical residues, : marks HEV-specific residues and blue letters represent unique moose HEV residues. (a) The X domain; underlined letters represent putative ADP ribose binding sites and green letters represent the active site. (b) The helicase domain; underlined letters represent putative ADP ribose binding sites and green letters represent Mg^2+^ binding sites. (c) The RNA dependent RNA polymerase domain; green letters represent the active site.

Alignment of the aa sequence of the moose HEV identified 164 residues corresponding to the characteristic putative X domain (macro domain), residues 795–958. When this region was blast searched, it matched most closely to gt4. Previous studies suggested that this domain has poly ADP-ribose-binding properties associated with replication and transcription (Egloff *et al.*, 2006; Neuvonen & Ahola, 2009). The seven characteristic X domain motifs, here designated motifs I–VII (Koonin *et al.*, 1992) were present in the moose HEV sequence (Fig. 1a). Potential ADP-binding and active sites were identified by the blastp. Upstream of motif I, two residues S805 and L806, present in moose HEV and in the gt1–4 strains, were according to blastp homologues to residues D and I, which have putative ribose binding properties. Motif I diverged with one residue from gt1–4 and was identical with rat, ferret and bat HEV. Motif II was unique to the moose HEV and diverged with only L837 compared to ferret HEV, while Motif III and IV remained conserved with the consensus sequence. Motif V contained three substitutions whereof L889 and I892 were unique making this motif more hydrophobic than in other HEVs, while motif VI diverged with F903 compared to the consensus sequence. Motif VI residues S-GIY908–911 appeared according to blastp as homologues to STGVY related to the putative ADP-binding site. Motif VII had three substitutions: two of them, V953 and M958, were partially shared with some animal HEVs, while K956 was unique to moose HEV.

#### Characterization of the RNA helicase.

The HEV RNA helicase has been shown to have NTPase and RNA unwinding activities and has been classified into the helicase superfamily SF-1 (Karpe & Lole, 2010a, b). The putative RNA helicase in the moose HEV was 233 aa corresponding to residues 971–1203. Nt and protein blast with this region showed the closest match with gt1. All six signature motifs typical for the helicase superfamilies were identified (Gorbalenya *et al.*, 1989) (Fig. 1b). Motif I of the moose HEV showed only a unique K991, followed by V993 substitution and eight conserved residues with nucleotide binding properties (Karpe & Lole, 2010b). Motif Ia contained one unique I1012 and a T1015 shared with avian and trout HEV. Motif II had one unique L1048, followed by the conserved residues DEAP1049–52, which have been shown to be involved in magnesium ion binding (Karpe & Lole, 2010b). Residues L1070 and Q1076 made motif III unique, while motif IV was identical to the consensus sequence. Two out of four residue substitutions, K1170 and V1173 were unique, making motif V most diverged among the six motifs. This motif was also highly divergent in the bat and trout HEV strains. In motif VI there were two residues divergent from the corresponding region of gt1–4-Uwb: R1198 that was shared with trout and avian HEV strains, and one unique V1200 resulting in an increased hydrophobicity of the motif.

#### Characterization of the RNA dependent RNA polymerase (RdRp).

The putative moose HEV RdRp was estimated to be formed of 488 aa, corresponding to aa positions 1218–1705 and a blastp search of this region resulted in a match with gt3 strains. The eight motifs in the HEV RdRp (Koonin *et al.*, 1992), were all present in the moose HEV RdRp (Fig. 1c). Motif I was highly conserved among the HEVs including in the moose HEV, with substitutions only in the avian and bat HEV strains. Two unique aas, M1430 and S1440, made the moose HEV motif II differ from the other HEVs. Among the motifs, motif III was most divergent with six residue substitutions in the moose HEV of which four were unique, A1456, N1458, P1461 and A1463. In the highly conserved motif IV the moose HEV had one unique substitution, D1490. Motifs V and VI were also highly conserved among the HEVs, with the active site GDD1570–72 situated in motif VI. Motif VII in moose HEV had two unique substitutions, D1610 and V1613, and motif VIII had one I1623 substitution.

### Identifying ORF2/3 start codons in the junction region (JR)

**Fig. 2.**
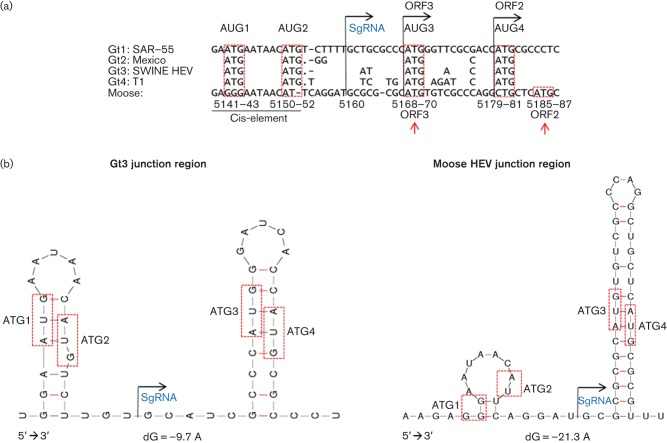
Identification of putative SgRNA with ORF2 and ORF3 start codons. (a) MSA of gt1–4 and moose HEV junction region. The SgRNA start position is indicated. The cis-element is underlined. Thr ORF2–3 start codons are marked, but with red arrows for moose HEV. (b) Mfold predicted two RNA secondary structures using gt3 or the moose HEV junction region. The ORF3 and ORF2 start codons were in the second stem structure of both HEVs. The ATG1–2 degeneration in moose HEV shown in (a) caused the truncated stem structure shown in (b). Position of ATG codons is shown by red dashed boxes.

The junction region of the HEV genome contains a cis-reactive element which may be the promoter for the 2 kb bi-cistronic SgRNA for ORF2 and ORF3 (Graff *et al.*, 2005). This region also contains loop structures important for virus replication (Cao *et al.*, 2010). The start of this putative SgRNA could be located at nucleotide position 5160 (Fig. 2a). Two start codons, designated AUG3 and AUG4, representing the authentic start codons for ORF3 and ORF2 (Huang *et al.*, 2007) were identified in the stem of the second stem–loop structure (Fig. 2a, b). Moose HEV had a different cis-reactive sequence element compared to other genotypes (Fig. 2a) and lacked the otherwise conserved AUG1 and AUG2. Mfold prediction of the cis-element revealed a first loop structure with truncated stem in the moose HEV (Fig. 2b). AUG3 was retained in the moose HEV, while AUG4 was three nucleotides downstream compared to AUG4 of gt1–4 strains, giving a longer stem length and a one nucleotide smaller loop (Fig. 2a, b). The SgRNA was estimated to be 2014 nt long, excluding the poly(A) sequence.

### Characterization of ORF2

ORF2, which encodes the viral capsid, was deduced to be at nt positions 5185–7152, and was in an alternative reading frame than ORF1. The 1968 nt sequence translated to a presumed 655 aa capsid protein, which was five aa shorter than ORF2 of gt1–4. ORF2 of the moose HEV had the highest nt (64.8–67.6 %) and aa (70.9–74 %) similarity to ORF 2 in gt1–4, rabbit HEV and unclassified wild boar HEV strains (Table 2). The moose HEV ORF2 had two aa deletions and four substitutions in the N-terminal signal sequence involved in translocating ORF2 to the ER (Ahmad *et al.*, 2011) (Table 3a, Fig. S3). The subsequent region was followed by a region of 12 arginines compared to 14 in the consensus sequence (Fig. S3) and most likely binds to the HEV RNA genome (Mori & Matsuura, 2011). This region was absent in trout HEV for unknown reasons. The crystal structure of the capsid protein has been separated into three domains, designated the shell (S), middle (M) and protruding (P) domains (Xing *et al.*, 2010). All three domains (Fig. S3) of the deduced moose HEV ORF2 were compared with the corresponding regions of the other HEV variants and showed several unique aa substitutions; four in the S domain, six substitutions, one G-deletion between residue position 404–405 in the M domain and 23 substitutions in the P domain (Table 3a). Three conserved asparagines representing potential glycosylation sites (Zafrullah *et al.*, 1999) were also identified in the moose HEV ORF2 (Table 3a). The stop codon of the moose HEV ORF2 was TAG, which was also observed in ferret and gt1–2 while the stop codon in gt4, Uwb and RAT HEV was TGA, and in gt3, bat, avian and trout, it was TAA.

**Table 3.**
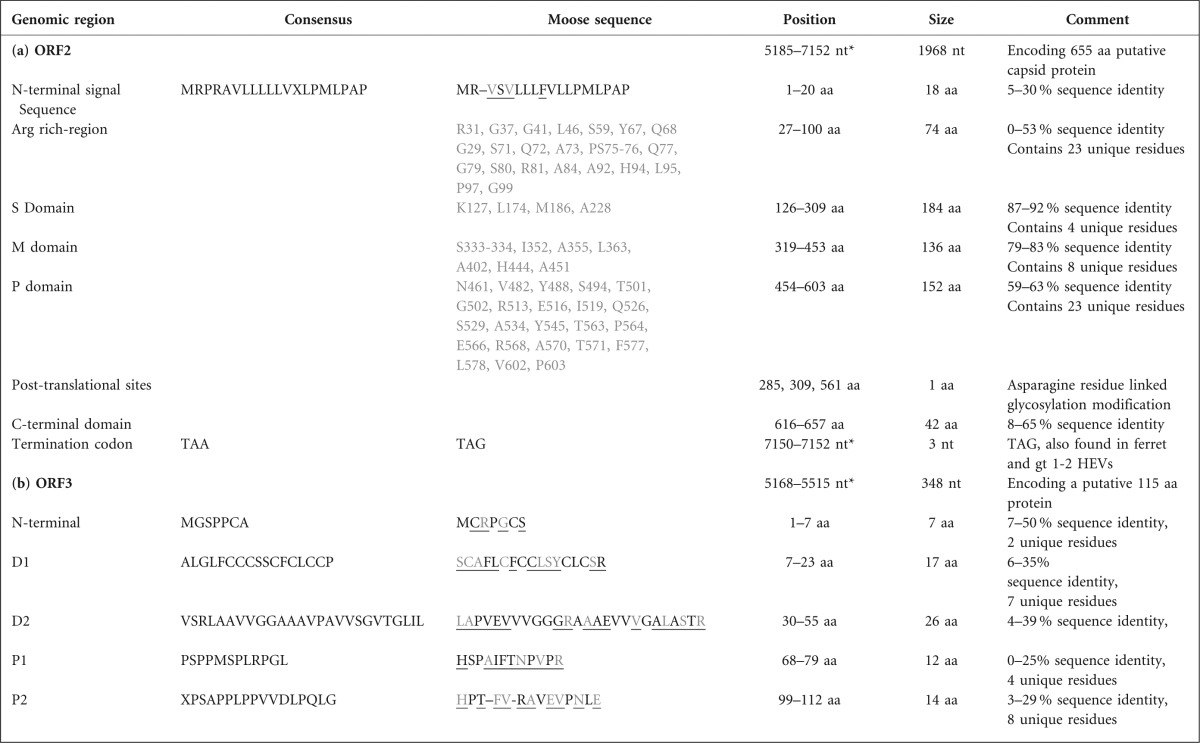
Moose HEV sequences. (a) ORF2 region with consensus sequence derived from multiple sequence alignment (Fig. S3) with strains taken from Table S1. Each region was derived from Mori & Matsuura (2011). (b) ORF3 properties with consensus sequence derived from aligned HEV strain sequences taken from Table S1. Each region was derived from Ahmad *et al.* (2011). In both (a) and (b), underlined residues indicate moose HEV substitutions while grey letters indicate unique residues for moose HEV

* Positions based on the reference gt3 SWX07-E1 (EU360977.1) genome.

### Characterization of ORF3

The putative ORF3 protein was identified by MSA and Mfold analysis to be at nt positions 5168–5515 and this 348 nt sequence corresponded to an 115 aa protein (Fig. 2, Table 3b). It showed nt (22–56 %) and aa (8–35 %) sequence similarity with other HEVs (Table 2). Four domains characterize ORF3 (Ahmad *et al.*, 2011) and all were present in the moose HEV (Table 3b). The highly diverged ORF3 N-terminal region contained two unique residues. The following hydrophobic domain 1 (D1) with a region of cystines has been associated with binding to the cytoskeleton, microtubules and mitogen-activated protein kinase (Kannan *et al.*, 2009; Kar-Roy *et al.*, 2004; Zafrullah *et al.*, 1997). The subsequent hydrophobic domain 2 (D2) has been associated with haemopexin binding (Ratra *et al.*, 2008), and both domains contained seven and eight unique residues. The two overlapping motifs PMSP and SPLR, as part of the P1 domain, acting as potential kinase substrates (Zafrullah *et al.*, 1997) were only observed in gt1 and some gt3s, while moose HEV displayed many unique residue substitutions making the target serine absent for possible kinase phosphorylation. The two overlapping PXXP motifs in the P2 domain, which are associated with SH3 protein domain binding and ORF3 interaction (Korkaya *et al.*, 2001), were detected in the gt1–4, but mutated in the moose HEV and in other animal HEVs.

### 3′UTR properties

The 3′UTR was 82 nt in length, spanned positions 7153–7227 and terminated with 26 nt poly(A). This region was highly diverged with 54 % sequence identity to gt3 SWX07-E1 and is thought to fold into stem–loop and hairpin structures and play a role in HEV replication (Ahmad *et al.*, 2011).

### The phylogenetic relationship of moose HEV with other HEVs

**Fig. 3.**
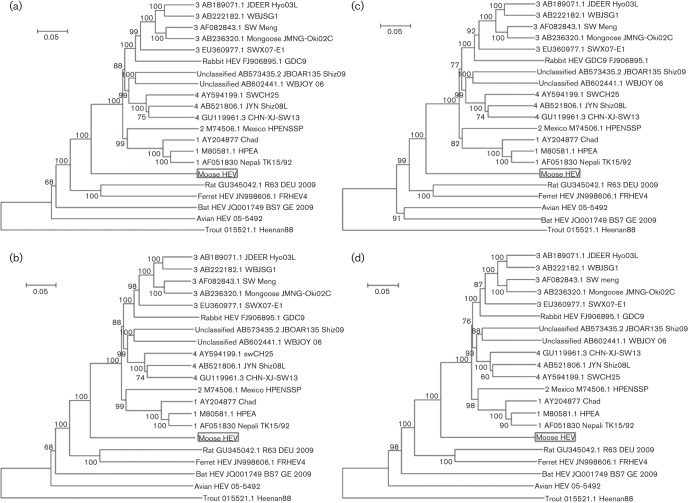
Phylogenetic nt relationship between HEVs based on a neighbour-joining approach using a Tamura-Nei model with estimated γ-parameter and 1000 bootstraps. (a) Concatenated ORF1 in-frame with ORF2, γ = 0.38. (b) Partial 2.16 kb ORF1 fused in-frame with ORF2, γ = 0.38. (c) Partial ORF1, γ = 0.42. (d) ORF2, γ = 0.3. Node numbers 1–4 represent gt1–4. Bar, genetic distance.

The moose HEV deviated in its own phylogenetic tree branch, sharing the same ancestor with the gt1–4-Uwb group. This occurred regardless if the generated phylogenetic trees were based on different regions in ORF1 or ORF2 or both ORFs combined (Fig. 3a–d). The 2.16 kb based phylogenetic tree (Fig. 3b) was similar to the 5.1 kb and ORF2 based trees (Fig. 3a, d), indicating that this region alone was sufficient for phylogenetic analysis. Only one phylogenetic tree (partial ORF1) was different, with a shared ancestor between avian and bat HEVs (Fig. 3c). An alternative method for investigating genetic relationships with other HEVs through aa p-distances (Smith *et al.*, 2013) was also evaluated. It resulted with the highest p-distance value of 0.83 when moose HEV was compared to trout HEV, while the values were between 0.22–0.52 for other HEV variants (Table 4). Higher p-distance values in animal HEVs corresponded well with the phylogenetic trees (Fig. 3a–d).

**Table 4.** Amino acid sequence percentage identity (lower left) and amino acid p-distance (upper right) relationship matrix of HEVs based on partial ORF1 in-frame fused with ORF2 with removed non-residue-coding junction region Moose HEV values are underlined separating the animal HEVs from Gt1–4-Unclassified wild boar 1–2 group. The p-distance is the proportion of amino acid sites at which the two sequences to be compared are different. It is obtained by dividing the number of amino acid differences by the total number of sites compared. The p-distance separating the genotypes is with a *P*-distance value of at least 0.06.

HEV variant	Gt 1	1	1	2	3	3	3	3	3	Rabbit	4	4	4	Uwb1	Uwb2	Moose	Ferret	Rat	Bat	Av	Trout
Gt 1 M80581.1 HPEA	.	0.02	0.01	0.09	0.09	0.10	0.10	0.09	0.09	0.11	0.09	0.10	0.10	0.11	0.12	0.28	0.41	0.43	0.48	0.51	0.82
Gt 1 AY204877 Chad	98.6	.	0.02	0.09	0.09	0.09	0.09	0.09	0.09	0.11	0.09	0.10	0.10	0.11	0.12	0.28	0.41	0.43	0.48	0.51	0.82
Gt 1 AF051830 Nepali TK15/92	99.0	98.0	.	0.09	0.10	0.10	0.10	0.10	0.10	0.11	0.10	0.10	0.11	0.12	0.12	0.28	0.41	0.43	0.49	0.51	0.82
Gt 2 M74506.1 Mexico HPENSSP	90.4	90.0	90.0	.	0.10	0.11	0.11	0.11	0.10	0.12	0.11	0.11	0.11	0.12	0.13	0.29	0.41	0.44	0.49	0.52	0.83
Gt 3 EU360977.1 SW07-E1	89.9	90.0	89.0	88.0	.	0.03	0.03	0.03	0.03	0.07	0.08	0.08	0.09	0.08	0.10	0.28	0.40	0.43	0.49	0.52	0.83
Gt 3 AB189071.1 JDEER-Hyo03L	89.2	89.0	89.0	88.0	96.0	.	0.01	0.01	0.02	0.07	0.08	0.08	0.08	0.09	0.10	0.29	0.40	0.43	0.49	0.51	0.83
Gt 3 AB222182.1 WbJSG1	89.2	89.0	89.0	88.0	96.0	98.0	.	0.01	0.02	0.06	0.08	0.08	0.09	0.09	0.11	0.29	0.40	0.43	0.49	0.52	0.83
Gt 3 AF082843.1 SW Meng	89.3	90.0	89.0	88.0	96.0	99.0	98.2	.	0.01	0.07	0.08	0.08	0.08	0.09	0.11	0.28	0.40	0.43	0.49	0.51	0.82
Gt 3 AB236320.1 Mongoose JMNG-Ok	89.7	90.0	89.0	88.0	96.0	98.0	98.2	98.6	.	0.06	0.08	0.08	0.08	0.09	0.11	0.29	0.40	0.43	0.49	0.51	0.83
Rabbit HEV FJ906895.1 GDC9	87.9	88.0	87.0	86.0	93.0	92.0	92.7	92.3	93.0	.	0.10	0.10	0.11	0.11	0.13	0.29	0.40	0.43	0.49	0.52	0.83
Gt 4 AB521806.1 JYN Shiz08L	89.2	89.0	89.0	87.0	91.0	91.0	90.9	91.0	91.0	88.3	.	0.02	0.03	0.08	0.10	0.28	0.40	0.42	0.49	0.51	0.83
Gt 4 AY594199.1 SWCH25	89.2	89.0	89.0	87.0	91.0	91.0	91.1	91.2	91.0	88.5	97.5	.	0.02	0.08	0.09	0.29	0.39	0.42	0.49	0.51	0.83
Gt 4 GU119961.3 CHN-XJ-SW13	88.8	89.0	88.0	87.0	91.0	91.0	90.5	90.9	91.0	88.2	97.1	97.0	.	0.09	0.10	0.29	0.40	0.43	0.49	0.52	0.83
Unclassified AB573435.2 JBOAR1 (UWB1)	87.4	88.0	87.0	86.0	90.0	90.0	89.6	90.0	90.0	87.8	90.7	91.0	90.4	.	0.09	0.28	0.40	0.43	0.48	0.51	0.82
Unclassified AB602441.1 WBJOY (UWB2)	86.6	86.0	86.0	86.0	88.0	88.0	88.1	88.1	88.0	85.5	89.3	90.0	89.4	89.0	.	0.29	0.39	0.42	0.49	0.52	0.83
Moose HEV	69.7	70.0	69.0	69.0	69.0	69.0	69.0	69.4	69.0	68.4	69.2	69.0	68.4	69.0	67.8	.	0.41	0.43	0.49	0.52	0.81
Ferret HEV JN998606.1 FRHEV4	56.7	56.0	56.0	56.0	57.0	57.0	57.1	57.4	57.0	56.8	56.9	57.0	57.0	57.0	57.2	55.8	.	0.24	0.48	0.52	0.83
Rat GU345042.1 R63 DEU 2009	54.6	54.0	54.0	54.0	54.0	55.0	54.7	54.9	55.0	54.4	55.1	55.0	54.9	55.0	55.0	54.8	73.9	.	0.51	0.54	0.82
Bat HEV JQ001749 BS7 GE 2009	48.2	48.0	48.0	47.0	47.0	48.0	47.6	47.7	48.0	47.1	47.9	48.0	47.6	48.0	47.4	47.5	48.8	46.0	.	0.50	0.83
Avian HEV 05-5492 (Av)	45.7	46.0	45.0	45.0	45.0	45.0	45.1	45.3	45.0	44.7	45.5	45.0	45.1	45.0	44.8	44.6	44.9	44.0	48.0	.	0.82
Trout HEV 015521.1 Heenan88	15.3	15.0	15.0	15.0	15.0	15.0	15.3	15.3	15.0	14.7	14.9	15.0	15.1	15.0	14.8	16.1	14.7	15.0	15.0	16.5	.

## Discussion

### A new member in the *Hepeviridae* family

In this study we describe, to the best of our knowledge, the first detection of a hepatitis E like virus in moose. Even though the sequences were highly divergent from known gt1–4, three HEV specific ORFs could be detected in the genome of this novel virus. The ORFs encode the non-structural proteins in ORF1, capsid protein in ORF2 and the multifunctional phospho-protein in ORF3. This observation together with ORF2/3 start codon and phylogenetic analysis (Figs 2 and 3), supported the classification of this new virus as a member of the *Hepeviridae* family. Even though there were several nucleotide substitutions in primer binding sites, real-time PCR assays were still able to detect the virus (Fig. S2). Future screening should benefit from redesigning the primers and probes for optimal detection. Primer pair 4 (Table 1) should in theory result in a larger HEV amplicon covering a larger sequence from the putative 5′ terminal methyltransferase to RdRp, but only a smaller amplicon was obtained starting from the putative proline hinge region. Although several primer pair combinations were tested, we were unsuccessful in amplifying the remaining moose HEV sequence at the 5′ end. This suggested an incomplete virus genome and may be due to degradation, or by high sequence divergence in combination with low virus concentration. The 2.16 kb amplicon (Table 1) was useful for phylogenetic characterization (Fig. 3b), since it covered regions in RdRp and ORF2 commonly used for HEV genotyping (Mizuo *et al.*, 2002; Zhai *et al.*, 2006). The secondary structure analysis of the JR (Fig. 2) should be valuable for identifying the ORF2/3 start codons in other uncharacterized novel divergent HEVs.

Four genotypes are recognized according to the ICTV *Hepeviridae* study group and there are still no consistent criteria for classification for novel HEVs. A recently introduced HEV species concept suggested that aa p-distances would provide an approach for evaluating taxonomic relationship (Smith *et al.*, 2013). The authors presented aa p-distances of 0.25–0.50 between HEV variants and as a comparison they used *Picornaviridae* classification that did not differ by more than 0.58 between genera (Knowles *et al.*, 2012). The p-distances of moose HEV did not surpass 0.58 and corresponded to an aa identity of 45–70 % to other HEVs (Table 4). The trout HEV was the only exception that surpassed 0.58 with values 0.81–0.83 corresponding to 15–17 % aa identity to other HEVs (Table 4). This was in line with the values of 0.60–0.90 observed by Smith *et al.* (2013). Therefore, the HEV genus cut off value may be in the 0.50–0.60 interval and adjusted by additional HEVs discovered in the future. We propose moose HEV as a new species, together with four other proposed species (HEV variants infecting humans and pigs; variants infecting rat and ferrets; variants from bat and those from poultry; Smith *et al.*, 2013) as members of the *Hepevirus* genus when using the cut off value of 0.58. The trout HEV with values >0.58, would then be classified as a member of a separate genus. More sequences from other moose HEV strains will confirm the separate phylogenetic branch deviation (Fig. 3a–d) of the moose HEV, and will show if moose HEV forms its own group consisting of separate genotypes, as has been shown for avian HEV (Bányai *et al.*, 2012; Bilic *et al.*, 2009; Sprygin *et al.*, 2012; Zhao *et al.*, 2010) and rat HEV (Li *et al.*, 2013).

### Host specificity, virulence and zoonotic aspects

The diverged moose HEV genome most likely reflects host adaptation, and sequenced regions that attract the most attention are ORF1; motifs I, II, V and VII of the X domain; motifs I, Ia, II, III, V, VI and VII of the helicase domain; and motifs II, III, IV and VII of the RdRp (Fig. 1a–c). These regions contain mutations compared to the consensus sequence and some are unique to the moose HEV. As a comparison, almost the entire of motif III in the RdRp of rubella virus was degenerate and even within the HEVs there was great aa diversity, implicating that this motif is suited to a more flexible adaptation (Fig. 1c). The D1461 in this motif was conserved in gt1–2, gt4 and in rabbit HEV. Gt1–2 and 4 are considered to be more pathogenic than gt3 (Abdelwahab *et al.*, 2012) and might share common potential virulence factors. Previous study of chimeric HEVs containing JR, ORF2 and 3′UTR from gt3 or 4 in the backbone of gt1 failed to establish infection in pigs, suggesting that the 5′UTR and ORF1 may also be involved in cross-species infection (Feagins *et al.*, 2011). The gt1 blast match of the moose HEV helicase domain may explain it as one factor by which ORF1 contributes to host restriction. Reinfecting pigs with the chimaeric gt1 backbone replaced with either the gt3 or gt4 helicase might confirm this hypothesis.

Previous aa substitution studies on certain aa pairs in the ORF2 P domain resulted in lack of virion attachment activity in HEV susceptible Huh7 and A549 cells (Yamashita *et al.*, 2009). A single hydrophobic aa substitution (T562V) in one of these aa pairs (aa pair T562 and N560) was found in moose HEV and bat HEV. Substitutions like this may disturb virion binding, leading to altered host specificity. The study of bat HEV observed that ORF3 in many animal HEVs was highly divergent (Drexler *et al.*, 2012), including the moose HEV (Tables 2 and 3b), and may reflect changes in host range and potential virulence properties. The 5′ terminal end region in moose HEV could not be analysed in this study and it would be interesting to detect other putative ORFs, like in ferret and rat (Johne *et al.*, 2010; Raj *et al.*, 2012).

In general, the detection of new viruses in animals usually raises the question regarding their zoonotic potential. Currently, the zoonotic transmission of HEV gt3–4 has been frequently reported, in association with the consumption of pig, wild boar and deer products in industrialized countries (Li *et al.*, 2005; Takahashi *et al.*, 2004; Tei *et al.*, 2003). Moose meat is consumed on a regular basis and therefore the zoonotic potential of HEV in moose is important to study. Experimental cross-species studies have been attempted with divergent HEVs like rat and avian HEV, but with negative results (Cossaboom *et al.*, 2012; Huang *et al.*, 2004), indicating limited host range. It is tempting to speculate that the moose HEV also has a narrow host range and features like a highly divergent genome indicates that this may be so. The application of cell culture and animal models could be useful for testing its cross-species transmission properties.

More divergent animal HEVs and gt1–4 that are more closely related to human HEV do exist in parallel leading to competition, but this also opens possibilities for recombination to occur within the host (Shukla *et al.*, 2011; Wang *et al.*, 2010). An example is the human gt1–2 vs gt 3–4 infections, and another example is the divergent rat HEV (Johne *et al.*, 2010) and the recently discovered rat gt3-like HEV (Lack *et al.*, 2012). Could similar evolution also occur in the Cervidae family, including in moose? The moose HEV may be the more divergent type with narrow host specificity, while the deer gt3 may represent the flexible variant with a wider host range. It would be interesting to see if this moose HEV could infect other species of Cervidae and if the deer gt3 could establish infection in moose. Previous studies revealed that gt3 HEVs are present in both domestic swine and wild boars in Scandinavia with high sequence similarity. HEV isolates taken from a Swedish patient revealed high sequence similarity with porcine HEV isolates. The strains appeared to be phylogenetically clustered into specific geographical clades; country- and even county-specific, thus opening the possibility to derive the geographical origin of HEV strains (Norder *et al.*, 2009; Widén *et al.*, 2011). A geographical clustering for rat HEV was observed in Germany, which when combined with serological analysis demonstrated antigenic differences between rat HEV and HEV gt3 antigen, indicating aa divergence in the immunogenic region corresponding to the P domain of ORF2 (Johne *et al.*, 2012). The moose HEV P domain showed 23 unique residues compared to other HEVs (Table 3a), indicating that antigenic differences might also exist. It would be interesting to investigate these observations in more detail for the moose HEV as well.

This HEV positive moose was emaciated and had an *Anaplasma phagocytophilum* infection in combination with other infections and infestations. What significance the infections had for the condition and myocardial injury of the moose is not possible to determine. Anaplasma infections are common tick-borne infections in animals and exhibit immunosuppressive properties (Rikihisa, 2011; Stuen, 2007).

In summary, more investigation is needed to better understand the infection biology, epidemiology and clinical manifestation of moose HEV in moose and humans. It is important that the public should be aware of that handling and consumption of HEV contaminated moose meat/organs may pose a risk for HEV infection.

## Methods

### 

#### Homogenization of liver samples.

Six Swedish moose samples taken from liver and/or kidney were homogenized in 2 ml grinding tubes (Eppendorf) containing 2 mm zirconia beads (BioSpec Products) and 600 µl buffer RLT from an RNeasy Mini kit (Qiagen).

#### RNA extraction and cDNA synthesis.

Total RNA was extracted from homogenized liver/kidney samples with an RNeasy Mini kit (Qiagen) according to the manufacturer’s instructions. The concentration and quality of RNA was determined by NanoDrop (NanoDrop Technologies). The 20 µl cDNA synthesis mix consisted of 1 µl Oligo dT_(20)_ (Invitrogen) or 1 µl GeneRACER Oligo dT_(24)_ (Invitrogen), used for priming cDNA synthesis with 3 µl RNA, 1 µl (40 U) of RNaseOUT (Invitrogen) and 1 µl (200 U) of Superscript III, RNase H^−^ reverse transcriptase (Invitrogen). One microlitre (end concentration 5 %) polymerase GC melt from an Advantage GC 2 polymerase mix kit (Clontech) was also added to facilitate amplification of high GC-content regions and reduce secondary structure formation in the HEV genome (Xia *et al.*, 2008). The cDNA reaction was kept at 50 °C for 60 min, followed by 15 min incubation at 70 °C. The reaction was finalized with 2 U of *Escherichia coli* RNase H (Invitrogen) for 20 min at 37 °C.

#### HEV real-time PCR and PCR assay.

Three microlitres of extracted and purified RNA from moose liver and kidney were analysed by real-time (RT)-PCR using an Ag-Path-ID one-step RT-PCR kit (Applied Biosystems), with a total volume of 12.5 µl containing 250 nM JVHEV forward respective reverse primers and 100 nM Cy5 based probe targeting the overlapping ORF2/3 region (Jothikumar *et al.*, 2006) and 0.4 × enzyme mix, or with primers targeting a sequence downstream of the ORF2 region with 500 nM forward and 250 nM reverse primers, 250 nM, 260 nM FAM based probe (Gyarmati *et al.*, 2007) and 1× enzyme mix. Both methods have been shown to be more sensitive in comparison with other HEV detection methods (Vasickova *et al.*, 2012) and combining both methods increases the chance of detecting clinical HEV positive samples. The samples were loaded into a Rotor-Gene 3000 (Corbett Research) with the following thermal steps: 45 °C for 10 min, followed by 95 °C for 15 min, cycled 55 times between 95 °C 15 s, 60 °C 60 s. Fluorescence was monitored during the annealing step of each cycle. The diluted full HEV genome of gt3 SWX07-E1 isolate cloned into a plasmid (Xia *et al.*, 2008) with a known concentration was used as a control and for the generation of standard curves.

To acquire more sequence information, we used PCR primer pair 1 (Table 1) targeting part of the RdRp giving a 383 nt fragment commonly used for genotyping (Zhai *et al.*, 2006) with 30 µl PCR mix: 6 µl of synthesized HEV positive cDNA template, 1.2U Platinum *Taq* polymerase (Invitrogen), 1× PCR RXN buffer, 1 mM MgCl_2_, 0.2 µM of each ESP and EAP primer, 5 % DMSO and 0.2 mM dNTP. The cycling parameters were 95 °C 3 min, cycled 40 times 94 °C 1 min, 55 °C 1 min, 72 °C 1 min and finishing with 72 °C for 10 min. To obtain more sequence information the forward primer ESP (Zhai *et al.*, 2006) and ORF2, HE041 reverse primer (Mizuo *et al.*, 2002) were used in conventional PCR. The PCR mix contained 1 µl cDNA as template with 0.15 µl Phusion Hot Start High-Fidelity DNA polymerase (Thermo Scientific, Finnzyme) with provided 1× GC buffer, 0.3 µl 0.2 mM dNTP, 0.5 µM ESP forward primer, 0.5 µM HE041 reverse primer and 0.45 µl DMSO (final 3 %) were also added in a total PCR volume of 15 µl. The PCR program had the following profile: 98 °C for 2 min, then cycled 40 times 98 °C 20 s, 65 °C 30 s, 72 °C 2 min and finished with 72 °C for 10 min. An overlap PCR primer walking strategy was used to further extend the 5′ side of the moose HEV genome by primer pair 4 (Table 1). The PCR profile was similar to that mentioned above, but with combined *T_m_* and extension temperature of 72 °C for 3 min.

All amplified PCR products were verified by agarose gel (LE Agarose, Semkem) electrophoresis in gels of different percentages depending on the fragment size.

#### Purification, cloning and sequencing of amplicons and RACE amplification.

The amplified putative HEV amplicons were purified according to the manufacturer’s instructions with a Wizard SV gel and PCR clean-up system (Promega) for larger amplicons and a PureLink Quick gel extraction kit (Invitrogen) for smaller amplicons. Size and single bands of purified amplicons were confirmed in agarose gels. The purified amplicons were sequenced by using same amplicon PCR primers in forward or reverse for confirming the HEV positive sequence. blast searches at both the nt and aa level was used to identify the sequenced amplicons. The phusion PCR generated products lacking 3′terminal overhangs required for TOPO XL cloning (Invitrogen). Therefore, poly(A) overhangs were synthesized before the cloning procedure in a 10 µl reaction mix with final concentration of 0.2 µM dATP, 1× PCR RXN buffer, 2.4 mM MgCl_2_, 0.5 U platinium taq polymerase (Invitrogen) and 8.22 µl of purified PCR product. The reaction was incubated in 72 °C for 15 min and put on ice according to the manufacturer’s instructions. The 3′UTR terminal end was amplified with primer pair 3 (Table 1) using a RACE kit (Invitrogen) resulted in a 1.3 kb overlapping PCR product according to PCR program profile 98 °C 2 min, 98 °C 10 s, 65 °C 30 s, 72 °C 2 min and 72 °C 10 min.

All sequencing reactions were carried out with a Big Dye Terminator Cycle Sequencing Ready reaction kit version 3.1 (Applied Biosystems) with program profile 95 °C 15 s, 50 °C 10 s, 60 °C 4 min cycling 25 times. Sequences were analysed in Lasergene 8 (dnastar). All primers for amplicon amplification and sequencing are referred to Table 1 and the papers of Koonin *et al.* (1992), Xing *et al.* (2010) and Ahmad *et al.* (2011) were used as guidance for identifying ORF1, ORF2 and ORF3 domain/motif regions.

#### Junction region analysis.

A multiple sequence alignment of JR containing the 5′-end, putative cis-reactive element and putative start codons for ORF2 and 3 were represented by AUG1–4 (Fig. 2). A secondary structure analysis with Mfold for the JR containing ORF2–3 start codons were investigated for a gt3 (AB481229.1) in parallel with the moose HEV with the similar approach as in Huang *et al.* (2007).

#### Phylogenetic analysis.

All phylogenetic trees (Fig. 3) and aa p-distance calculation (Table 4) was performed with mega 5.0 (Tamura *et al.*, 2011). Multiple HEV sequences (Table S1), were aa aligned and reconverted back to nucleotides. The Tamura-Nei evolutionary distance model was used for generating neighbour-joining phylogenetic trees with 0.30–0.42 γ-parameter values and 1000 bootstraps.
